# Three-dimensional computed tomography reconstruction in the era of digital personalized medicine

**DOI:** 10.1590/1516-3180.2022.14111125082022

**Published:** 2022-11-28

**Affiliations:** I MD. Attending Physician, Thoracic Surgery Service, Instituto do Câncer do Estado de São Paulo, Hospital das Clinicas HCFMUSP, Faculdade de Medicina, Universidade de Sao Paulo, Sao Paulo, SP, BR.; II MD, PhD. Full Professor, Thoracic Surgery Program, Instituto do Coração (InCor), Hospital das Clinicas HCFMUSP, Faculdade de Medicina, Universidade de Sao Paulo, Sao Paulo, SP, BR; Director, Scientific Department, Associação Paulista de Medicina (APM), São Paulo (SP), Brazil.; Associação Paulista de Medicina, Scientific Department, São Paulo, SP, Brazil

The radiological evaluation of anatomical structures is a fundamental part of the diagnostic and therapeutic workup in modern medicine. Since the discovery of X-rays by Wilhelm Conrad Roentgen in 1895, the medical community has added to its arsenal of instruments capable of evaluating the interior of the human body non-invasively.^1^ Each development being safer and more efficient radiological exams became capable of identifying, isolating, and analyzing—with high realism—the anatomy and functionality of organs, gaining popularity at almost the same rate at which they evolved.^2^

Computational imaging tests, most notably computed tomography (CT), have contributed significantly to the spread of image-based medicine. Since its invention in 1972 by Cormack and Hounsfield, the quality of tomographic images has progressed exponentially, driven by advances in informatics and computing science.^3^ The final product of a CT scan depends less on human influence in the image acquisition, and well-established protocols can be executed by professionals with a technical level to obtain adequate results. Unlike ultrasound examinations, which require a trained medical professional to perform an evaluation in real-time, tomographic images can be stored and evaluated by multiple professionals without limitations in terms of time or physical access to the patient. This quality facilitated its dissemination and allowed physicians from different areas and without previous in-depth radiology training, through repeated contact, to perform evaluations efficiently. However, its detailed analysis still lacks specialized workstations that allow the visualization of two-dimensional images in multiple planes, advanced contrast manipulation, image suppression techniques, and specific training in the field of medical radiology.

To address the difficulties in the analysis of bidimensional and monochromatic tomographic images, the technological evolution of graphic manipulation methods is being increasingly applied to the medical field.^4^

The study of three-dimensional (3D) rendering techniques for CT scans began in the 1970s, driven by the development of computer hardware and software. The dissemination of examinations performed on multislice helical CT scanners resulted in the generation of high-quality images with sufficient data for more complex and detailed renderings for a larger number of users. In this manner, techniques such as maximum intensity projection and surface shaded visualization were developed. However, limited by the technological development of data processing, these techniques use only 10% of the data available in the exams, using only part of the images to perform a low-quality 3D reconstruction; thus, this limits their applicability to certain circumstances.^4^

With the evolution of data processing capacity of modern computers, techniques such as volumetric rendering (VTR) could flourish to provide the medical community 3D images of CT scans with satisfactory quality.^5^ Despite high resolution images, VTRs cannot efficiently isolate anatomical structures. The final product works like a sculpture with parts that can, in some cases, be cut or erased, but without natural segmentation and individualization of the human body. Although it does not interfere with the evaluation of superficial and bony structures, it is an important limitation for the evaluation of deep structures and those that are proximal to other parts with similar densities.

To overcome this deficiency, the segmentation of anatomical structures (organs and tissues) can be performed using software and techniques specialized in extracting these data from CT scan. This technique uses computational power to differentiate countless shades of gray within the monochromatic spectrum of tomography and, based on Hounsfield units and structure contour, separates organs and tissues individually with high precision.^6^ Thus, personalized 3D graphic representations can be obtained, considering the unique anatomy of each patient. These virtual models can be manipulated in different ways at different angles, bringing the results obtained from the examinations to the actual spatial reality. This led to the development of a software industry specialized in the segmentation and post-processing of medical images, enabling integration with new technologies and opening the doors of the era of digital and personalized medicine.

With more consolidated applicability in areas such as orthopedics, facial surgery, and neurosurgery, the use of 3D reconstruction and CT segmentation has been evolving at great strides in surgical aid techniques.^7–9^ Solid organs, with little anatomical variations and simple vascularization, have benefited quickly because their rendering and segmentation require less complex software and algorithms. However, semi-automatic segmentation techniques are already available, allowing physicians with basic training to perform complex reconstructions such as vascular, hepatic, or pulmonary with just a few clicks. This automates and simplifies the acquisition of 3D reconstructions, which is essential for the dissemination of this technique. A simplified facility allows better multi-professional communication between surgeons, radiologists, and clinicians.^9,10^ The interdisciplinarity optimizes the results obtained by adding different points of view from the surgical technical vision to the anatomical radiological evaluation and its clinical implications.

In thoracic surgery, the evolution of lung resection techniques through the spread of sublobar resections for the treatment of benign diseases and lung cancer has boosted the use of 3D reconstruction in this field. Approximately more than half of the lung resections are performed in a minimally invasive manner, that is, through small incisions with the aid of optics and video material to visualize the intrathoracic structures. This type of surgery minimizes the chance of extensive handling of anatomical structures by the surgeon, limiting the ability to identify them by touch.^11^ In this context, the preoperative study regarding the vascular and bronchial structures to be treated becomes essential. The use of 3D reconstruction of chest CT scans facilitates this process and has shown positive results in reducing surgical time and intraoperative blood loss.^12,13^ Moreover, with the dissemination of robotic platforms in different specialties, the integration of these technologies has become increasingly feasible. The presence of a digital machine between the surgeon and the patient allows the application of technologies such as intraoperative augmented reality, which is capable of interposing 3D images to the surgical field, thus facilitating decision-making and increasing surgical precision.^14,15^ This integration allows us to envision AI-based functionalities capable of recognizing inaccurate and even dangerous surgical movements, thus reducing the rate of intraoperative accidents.

Extended reality, including virtual reality and augmented reality, is a technique capable of bringing 3D models even more into the real world. To provide a unique immersion sensation with binocular vision and ultra-realistic depth and visual texture sensations, they can be precisely employed in surgical planning and teaching for surgeons in the initial learning curve. It is no longer necessary to imagine the spatial distribution of the structures visualized in CT, allowing an easier understanding of the anatomy. Extended reality techniques are widely used in the field of endoscopic diagnosis. Virtual bronchoscopy, with the reconstruction of the tracheobronchial tree, identification of tumors and stenosis, and optimization of navigation techniques for performing biopsies of pulmonary nodules, are constantly expanding techniques.^15^ These 3D reconstructions can be used in virtual reality environments for realistic simulations, allowing the surgical team to perform simulated training like those employed in aviation.^16^ For example, surgeons and technicians can use 3D models and their customized features to simulate the best surgical approach and identify the best prosthesis or position for it to be used.

In addition, rendered images can be used for 3D printing. Because of the popularization and falling prices of printers and printing supplies, the construction of physical models helps surgeons from various specialties realistically experience what will be found in the surgery. Its use goes beyond preoperative planning and is also useful for patient counseling, teaching, and the development of bioimplantable materials. For example, in thoracic surgery, the construction of customized prostheses for chest wall and airway reconstructions is actively expanding.^17^ Still limited by manufacturing costs, 3D printing of customized prostheses in biocompatible materials such as polymethylmethacrylate and laser cutting of titanium structures reduce complications and optimize esthetic results.^18^ These techniques are in line with the growing concept of personalized medicine, considering the uniqueness of each individual pathology in the particular physiology of each human being.

Thus, 3D reconstruction technology based on CT scans shows promise in several fields. In the era of personalized medicine and the digitalization of medical care processes, its applicability will be broad, improving care results and academic performance. As discussed in this article, it is not an ultimate technology but a technological device that will serve as a basis for the development of several applications involving robotic platforms, artificial intelligence, extended reality, and 3D printing. The implementation of this technology in all institutional departments is essential in laying the groundwork for these future technologies and the benefits they can bring to patients and the scientific community.
